# A Review of Research on SLAM Technology Based on the Fusion of LiDAR and Vision

**DOI:** 10.3390/s25051447

**Published:** 2025-02-27

**Authors:** Peng Chen, Xinyu Zhao, Lina Zeng, Luxinyu Liu, Shengjie Liu, Li Sun, Zaijin Li, Hao Chen, Guojun Liu, Zhongliang Qiao, Yi Qu, Dongxin Xu, Lianhe Li, Lin Li

**Affiliations:** 1 College of Physics and Electronic Engineering, Hainan Normal University, Haikou 571158, China; 202206071306@hainnu.edu.cn (P.C.); 202206071348@hainnu.edu.cn (X.Z.); zenglina@hainnu.edu.cn (L.Z.); 202206071328@hainnu.edu.cn (L.L.); liushengjie@hainnu.edu.cn (S.L.); lisun@hainnu.edu.cn (L.S.); lizaijin@hainnu.edu.cn (Z.L.); chenhao@hainnu.edu.cn (H.C.); 068006@hainnu.edu.cn (G.L.); qzhl060910@hainnu.edu.cn (Z.Q.); quyi@hainnu.edu.cn (Y.Q.); 2 Key Laboratory of Laser Technology and Optoelectronic Functional Materials of Hainan Province, Hainan Normal University, Haikou 571158, China; jilinchangchun@yeah.net; 3 Hainan International Joint Research Center for Semiconductor Lasers, Hainan Normal University, Haikou 571158, China; 910335@hainnu.edu.cn

**Keywords:** LiDAR, vision sensors, SLAM, data fusion, autonomous navigation, dynamic environments, deep learning, semantic information

## Abstract

In recent years, simultaneous localization and mapping with the fusion of LiDAR and vision fusion has gained extensive attention in the field of autonomous navigation and environment sensing. However, its limitations in feature-scarce (low-texture, repetitive structure) environmental scenarios and dynamic environments have prompted researchers to investigate the use of combining LiDAR with other sensors, particularly the effective fusion with vision sensors. This technique has proven to be highly effective in handling a variety of situations by fusing deep learning with adaptive algorithms. LiDAR excels in complex environments, with its ability to acquire high-precision 3D spatial information, especially when dealing with complex and dynamic environments with high reliability. This paper analyzes the research status, including the main research results and findings, of the early single-sensor SLAM technology and the current stage of LiDAR and vision fusion SLAM. Specific solutions for current problems (complexity of data fusion, computational burden and real-time performance, multi-scenario data processing, etc.) are examined by categorizing and summarizing the body of the extant literature and, at the same time, discussing the trends and limitations of the current research by categorizing and summarizing the existing literature, as well as looks forward to the future research directions, including multi-sensor fusion, optimization of algorithms, improvement of real-time performance, and expansion of application scenarios. This review aims to provide guidelines and insights for the development of SLAM technology for LiDAR and vision fusion, with a view to providing a reference for further SLAM technology research.

## 1. Introduction

The application of LiDAR and vision fusion in SLAM (simultaneous localization and mapping) technology has become an important research direction in the field of autonomous navigation and environment perception. With the rapid development of autonomous systems, such as mobile robots, unmanned vehicles, and UAVs (unmanned aerial vehicles), the demand for environment-sensing and autonomous navigation capabilities has become stronger, and the combination of LiDAR and vision sensors would bring new opportunities for the development of SLAM technology. The performance and resilience of SLAM systems in complicated situations can be greatly enhanced by combining the data from vision sensors, which can provide rich environment texture information, with lidar, which can provide high-precision distance information. Some outstanding framework algorithms have been used in many sectors, with outstanding performance in the current stage of visual SLAM approaches [1], which is crucial for the safe navigation of autonomous vehicles in intricate urban settings [2]. Furthermore, when confronted with unfamiliar environments, SLAM technology enables robots to synchronize the creation of maps and the acquisition of their own position data, achieving efficient navigation—a crucial function in situations where GPS signals are limited or nonexistent.

The fundamental idea behind SLAM technology is to use sensors (such as LiDAR and vision sensors) to gather environmental data, a process that uses data algorithms and updates the device’s location and map in real time. The two concurrent tasks of localization and map creation form the basis of the technology. While mapping is the process of creating a map of the environment, localization is the process of estimating the device’s position in the environment using sensor data. Processing sensor input and combining information is essential to SLAM implementation. There are three types of SLAM: laser SLAM, vision SLAM, and a combination of the two, depending on the sensors.

Since LiDAR is a high-precision environment-sensing tool, laser SLAM can demonstrate its distinct advantages in complex environments with varying lighting and dynamic conditions. However, its limitations are also evident. For instance, its performance will deteriorate in feature-sparse environments with low texture and repetitive structures. Because laser data only contains distance information and no environmental texture details, it will be difficult to create maps in areas with few features. However, laser SLAM is also extremely dependent upon the environment and will suffer data sparsity issues in complex or dynamic situations (like congested cities). Similarly, it will result in data loss or poor judgment when identifying highly reflective or clear surfaces. Additionally, the LiDAR data typically lacks rich semantic information, making it difficult to provide object classification and recognition. The application of laser SLAM will be severely limited due to the massive point cloud data generated by the technology, which has high requirements for real-time processing and storage, particularly in dynamic environments where the demand for real-time performance and computational resources increases significantly. However, visual SLAM is more sensitive to changes in lighting and texture, and it performs poorly in scenes that are highly dynamic or have low light levels. Dynamic objects can interfere with visual SLAM in dynamic surroundings (e.g., pedestrians, automobiles, etc.). Typically, this approach depends on the motion model (such as translation and rotation) of the camera. Fast or complex motion may cause the model’s accuracy to drop, which could impact positioning accuracy and ultimately result in tracking failure or significant positioning errors; in low-light scenes, the reliability of feature extraction and matching will significantly drop due to the significant decline in image quality, which could result in positioning failure. On the other hand, visual SLAM is more affordable, has a higher degree of environmental adaptability with rich spatial color information and high resolution, and can better identify and distinguish both static and dynamic objects [3].

Thus, the combined usage of LiDAR and vision sensors overcomes their individual limitations and shows improved accuracy and more environmental adaptability in map creation and self-localization activities to handle more difficult application scenarios. Specifically, it can greatly increase the system’s adaptability and dependability when handling moving impediments in intricate and dynamic scenarios [4]. For instance, Young-Sik Shin et al. (2018)’s direct-vision SLAM method combining sparse depth information shows that integrating LiDAR and vision camera data can enhance accuracy and real-time performance [5].

To sum up, as SLAM technology continues to advance, the combination of LiDAR and vision sensors not only advances SLAM technology but also advances related fields of study and creates new opportunities for autonomous navigation and environment perception [6]. The theoretical foundation and practical direction for the SLAM system with increased efficiency and resilience achieved by the merging of LiDAR and vision SLAM technologies will be crucial in light of the current, increasingly complicated, application scenarios.

## 2. The History of SLAM Technology Development

**Figure 1:**
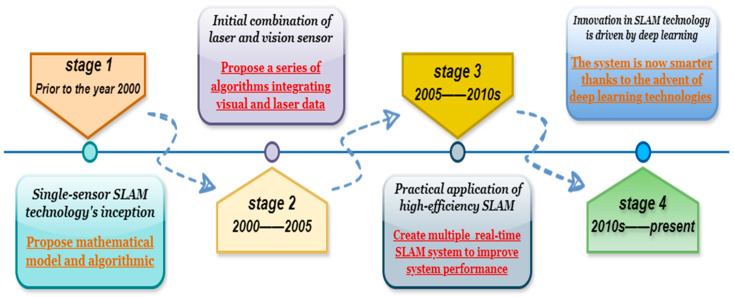
Overview of the SLAM research progress.

From early simple algorithms to modern complicated fusion technologies, SLAM technology has undergone multiple stages of development, creating a broad research area. At first, SLAM mostly depended on pose estimation and matching sparse feature points (Davison et al., 2007) [7]. Researchers have suggested many algorithm frameworks to maximize the performance of SLAM systems as computing power has increased. For instance, the effectiveness of SLAM algorithms is objectively assessed by measurements based on relative pose relationships, thereby correcting pose information and optimizing the map [8], avoiding the drawbacks of relying on a global reference frame. Combining multi-dimensional data gathered by additional sensors to optimize the overall SLAM system offers more benefits than improving relative pose algorithms. For instance, global trajectory consistency is optimized by combining the benefits of visual characteristics and 3D depth sensor scale information to simultaneously estimate the camera’s trajectory and create a detailed 3D model of the surroundings [9]. At the same time, algorithm frameworks for assessing the global pose error of SLAM systems and the drift of visual odometry systems have also been provided in order to facilitate objective comparisons between various algorithms [10], as shown in Figure 1. Prior to 2000, LiDAR (Lidar) was the primary tool used by SLAM technology for map creation and positioning. The extended Kalman filter (EKF) was frequently employed for state estimation in early algorithms, which was primarily reliant on a single sensor, especially laser scanners. This approach works well in small-scale settings but is not very flexible in large-scale ones. As processing power increased between 2000 and 2005, the SLAM algorithm started progressively developing, including visual data, embraced graph optimization and multi-sensor fusion techniques, and increased the system’s accuracy and resilience. Between 2005 and 2010, SLAM technology advanced quickly, with research concentrating on adaptive and real-time performance. A variety of real-time SLAM frameworks were also introduced, along with sparse features (like SIFT and SURF) for map building and global optimization methods (like bundle adjustment) to increase accuracy. Since 2010, the integration of laser and vision SLAM has advanced to a new level due to the advancements in deep learning and big-data technologies. The system’s performance is greatly enhanced through feature extraction and data association made possible by deep learning. It has improved the intelligence level and maintained a healthy balance between flexibility and real-time performance.

**Figure 2:**
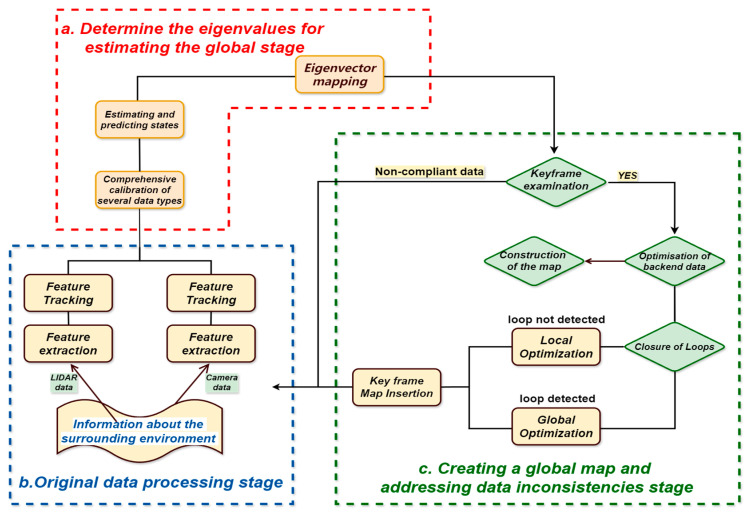
The three primary steps of framework for traditional SLAM architecture: (**a**) the eigenvalue determination for estimating the global stage; (**b**) Original data processing stage; (**c**) Global map creation and data inconsistencies stage.

Researchers have started investigating SLAM techniques based on dense visual information with the combined use of visual sensors. By densely modeling the environment, these techniques can further enhance the consistency and quality of map construction [11]. As a result, LiDAR and vision fusion technology have steadily gained popularity in SLAM research. Traditional SLAM technology typically assumes a static environment, but moving objects, in reality, can have a significant impact on positioning. This makes it different from early SLAM technology, in that it lacks stable robustness and has low flexibility and accuracy in the application process due to the strong influence of environmental factors. For instance, unfavorable weather circumstances (such as rain and fog) can produce more ambient noise for the SLAM system, or it cannot reliably extract features under complex illumination situations, which can result in low system accuracy. Effectively combining LiDAR and visual sensor data can greatly enhance SLAM’s overall performance, particularly its resilience in situations with quick motion and inadequate lighting [12]. Thus, the accuracy of the positioning of SLAM systems in complex environments has been greatly enhanced by the methods of scene segmentation based on semantic information of the environment and consistency check of dynamic objects; a SLAM framework based on the fusion of low-cost LiDAR and visual sensors (RGB-D camera) has been proposed [13] by building a novel cost function and combining the scanned image data, further improving the positioning accuracy. Researchers have shown the potential of visual sensors in complicated situations and configured a range of sensors to achieve high-precision visual and visual–inertial SLAM [14], as shown in Figure 2. The framework comprises three primary steps and adheres to the traditional SLAM architecture: (a) Determine the eigenvalues for estimating the global stage, by predicting the global stage’s eigenvalues, the SLAM system may strike a solid compromise between precision and real-time performance; (b) Original data processing stage, the original data processing has increased the SLAM system’s performance in complicated and dynamic contexts in addition to improving the data’s quality and dependability; (c) Creating a global map and addressing data inconsistencies stage. Resolving data inconsistencies and creating a global map can help to increase the map’s accuracy and consistency while lessening the effects of environmental changes and sensor mistakes.

Furthermore, laser SLAM in conjunction with deep learning can significantly improve the system’s performance in closed-loop detection, thereby showcasing the promise of fusion technology to increase the resilience of SLAM systems [15]. Performance in complicated situations can be effectively enhanced by employing recurrent neural networks (RNN) and convolutional neural networks (CNN) [16]. In addition, the dense visual GS-SLAM approach with 3D Gaussian representation can better balance accuracy and efficiency. The use of the GS-SLAM technique has greatly enhanced the quality and consistency of map building when compared to the prior real-time dense SLAM system based on surfels [17].

In summary, as computing power has increased and research continues to advance, current SLAM technology has evolved toward multi-sensor data fusion, particularly the use of laser radar and visual fusion SLAM technology, which is also constantly investigated and improved toward efficiency, accuracy, and robustness [18]. In addition to outlining the benefits and drawbacks of the technology, this paper evaluates and summarizes the state of research on laser and visual fusion SLAM technology and explains why laser radar and visual fusion are necessary.

## 3. The Primary Techniques for SLAM Technology Study

Both the durability of the system and the accuracy of map building have substantially increased with the integration of LiDAR and optical sensors. This combination successfully handles dynamic changes in complicated circumstances and is crucial for current applications [19]. Nowadays, SLAM technology that combines optical and LiDAR sensors has evolved into a variety of methods. The features, benefits, and difficulties of each fusion technique are explained in this study. Additionally, it draws attention to the similarities between these approaches and suggests fresh lines of inquiry for the advancement of this integrated SLAM system.

### 3.1. A Multimodal Data-Based Fusion Technique

**Figure 3:**
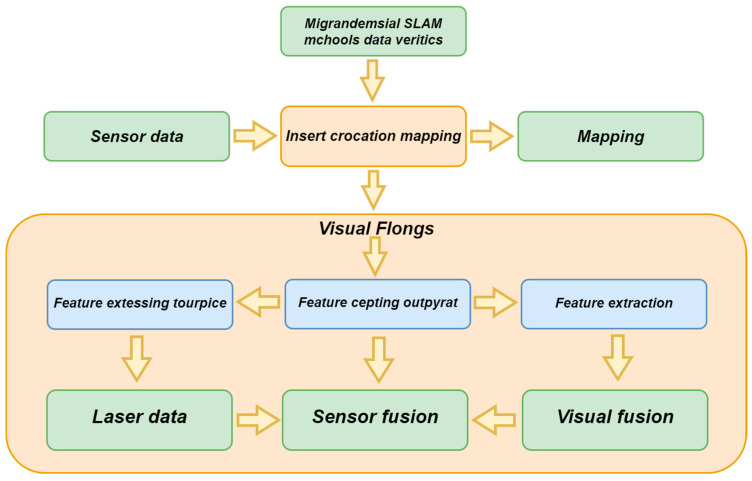
Diagram of the flow of multimodal data fusion technology.

In the SLAM technology of laser and vision fusion, the technical route of multimodal data fusion includes multiple interrelated steps, as shown in Figure 3. First, migratory SLAM uses existing knowledge to quickly adapt to the new environment and lays the foundation for the entire process. Next, multimodal data verification ensures consistency between the laser and visual data, providing reliable input for subsequent insertion interleaving mapping, which enhances map details. Visual flow analyzes the motion between adjacent frames to provide support for feature extraction, which extracts key feature points from laser and visual data, laying the foundation for integrating unified descriptors in the feature capture output stage. Laser data provides high-precision geometric information to assist in feature extraction. Subsequently, sensor fusion integrates data from different sources to improve overall positioning accuracy, while visual fusion integrates multiple image information to form a consistent environmental representation. Through the close connection of these steps, multimodal data fusion technology significantly improves the performance and robustness of SLAM systems in complex environments.

The merging of various sensor inputs and the optimization of the algorithmic framework are the main topics of current research on multimodal SLAM techniques. According to Kolar et al. (2020), who studied a range of fusion strategies in their research, merging data from LiDAR and vision sensors can greatly enhance autonomous navigation performance [20]. The LIO (laser-inertial odometry) and VIO (visual–inertial odometry) subsystems, which jointly estimate the system state by fusing their respective laser point cloud information or vision data with the IMU data, have also been proposed by algorithm researchers as part of the compact FAST-LIVO framework. They employ a direct photometric error minimization method to avoid the time-consuming feature extraction and matching process, achieve high-precision state estimation and map construction, and improve computational efficiency [21]. Better autonomous navigation performance under a range of environmental situations would be made possible by the multi-constraint optimal estimation framework developed by utilizing LiDAR information as visual features that are tightly connected [22].

Additionally, by closely integrating the target-tracking data with the SLAM system, researchers have been able to greatly enhance the overall system performance. The BEVFusion framework, which Liang et al. (2022) proposed, shows how vision and LiDAR information can be fused. It addresses the ability of vision information to continue performing the task on its own in the event of LiDAR failure, which is crucial for real-world applications [23]. The truncated symbolic distance field (TSDF) method, which has a much faster convergence rate and can greatly increase the accuracy of the camera localization and 3D reconstruction, as well as provide higher quality reconstruction results, is used to process the data in dense SLAM scenarios by combining the most recent neural radiation field (NeRF) technology with the SLAM system [24].

Consequently, the multimodal SLAM technique, which combines various sensor inputs, can increase the SLAM system’s accuracy, as well as its adaptability to challenging situations. For instance, several of the existing techniques, including ROVIO (robust visual–inertial odometry), VINS-Mono, and LOAM (lidar odometry and mapping), have demonstrated good performance and adaptability. For odometry and map updating, the LOAM method divides point cloud data into ground features and non-ground features, respectively. Through the use of feature matching for posture estimation, quick point cloud processing, and global optimization, this technique employs a hierarchical processing approach to increase map accuracy. The VINS-Mono algorithm is a visual–inertial navigation system that integrates data from inertial measurement units (IMUs) and monocular cameras. Using a sliding mode filter (SMF) and nonlinear optimization, it can conduct state estimation, enabling the real-time tracking of camera position and motion state. The extended Kalman filter (EKF) framework is used by the ROVIO algorithm, which combines visual feature tracking and IMU measurements to provide accuracy and robustness in dynamic situations. In general, multimodal data fusion algorithms exhibit broad applicability and strong feasibility, are more accurate in multi-sensor feature extraction and environmental modeling, and can integrate data from multiple sensors to enhance system robustness in complex environments. They also perform well in a variety of application scenarios, such as complex natural scenes, indoor navigation, and urban environments. The multimodal data fusion approach, on the other hand, focuses not only on how to gather and process data from various sensors but also on how to create an algorithm that can effectively combine the data, allowing the system to increase accuracy and have a stronger environment-sensing capability to handle increasingly complex scenes.

### 3.2. Methods of Feature-Based Fusion

It is common practice to use feature points for LiDAR and vision fusion in SLAM algorithms. For more precise localization and map creation, these techniques typically rely on feature points that are taken from sensors. Li et al. (2020) claimed that the system’s robustness in complicated situations is greatly increased by using the HF-Net model to extract local features (key points and descriptors) and global features (image descriptors and objects’ color, texture, and other attributes are referred to as image descriptors. These attributes can be used to summarize an image’s overall qualities and are appropriate for scene recognition and matching), particularly in terms of re-localization and closed-loop detection [25]. This method has the advantage of being able to make use of LiDAR’s high precision and richness of visual features, which allows for continued good performance in challenging scenarios.

Furthermore, the simultaneous acquisition of color and depth data using RGB-D sensors significantly increases the map’s accuracy and density [26]. Combining RGB-D SLAM with LiDAR allows for more efficient feature extraction and target tracking in complicated situations by optimizing the camera’s trajectory estimation using the precise information of LiDAR [27,28].

**Figure 4:**
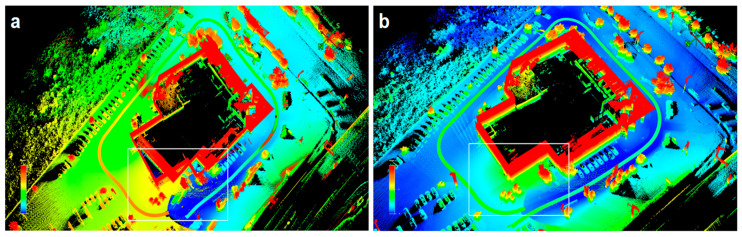
(**a**) The PVL-Cartographer system’s misalignment is depicted without closed-loop detection; (**b**) the outcomes following closed-loop detection. In (**a**,**b**), the region where loop closure detection is carried out is enclosed by the white rectangle. In order to merge LiDAR point clouds with panoramic photos, the system combines tilt-mounted LiDAR, panoramic cameras, and IMU sensors. It then uses internal data and algorithms to calculate the actual scale of the environment without the need for extra positional information. Even in surroundings with limited features, it may function effectively and dependably by achieving the seamless integration of data from many sensors, boosting the positioning and mapping results’ accuracy and dependability [30].

Through a deep integration of depth laser data and picture attributes, the deep fusion approach considerably improves the accuracy of 3D object detection [29]. By fusing panoramic photos with LiDAR data, Zhang et al.’s (2023) PVL-Cartographer approach tackles the problem of high-precision location and map creation in landscapes with few characteristics [30], as shown in Figure 4.

### 3.3. SLAM Technique with Semantic Information Assistance

Semantic information-assisted SLAM approaches boost the processing power of dynamic objects by incorporating semantic segmentation information. This approach commonly includes artificial intelligence, identifying objects in the scene to improve the flexibility of the SLAM system to changing situations [31]. In order to avoid the issue of tracking threads waiting for semantic information and maintaining the algorithm’s real-time performance, Liu et al. (2021) proposed a real-time visual dynamic RDS-SLAM algorithm that selects keyframes by adding semantic threads and semantic-based optimization threads [32]. This allows various speed segmentation methods to obtain the most recent semantic information for unified use. This approach has the benefit of being able to create maps and track reliably in dynamic real-time contexts. The system’s overall performance can also be improved by utilizing the benefits of neural implicit representation to identify and process dynamic objects with semantic information.

**Figure 5:**
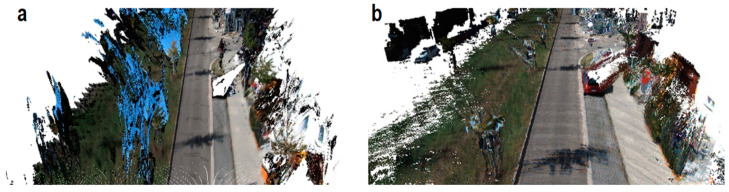
Comparison of the Lou et al. scheme and the quality of 3D reconstruction using DynaSLAM techniques: (**a**) reconstruction of DynaSLAM, (**b**) reconstruction of Lou et al.’s method [32].

Lou et al. (2023) suggested a SLAM technique based on the combination of LiDAR and monocular vision in order to get over the drawbacks that a single sensor faces in dynamic situations. The technique greatly increased positioning accuracy by using parallel threads to process low-resolution 3D point clouds and semantic pictures. The efficiency of LiDAR and vision fusion is demonstrated by the experimental findings, which show that the positioning error of this method has dropped by approximately 87% on several datasets. This suggests that the technology has the potential to increase positioning accuracy in complicated scenes [33], as shown in Figure 5. As a result, the semantic information-assisted SLAM approach offers numerous benefits, including increased resilience, better positioning accuracy, and support for challenging jobs.

Deep-learning processing of the target is, therefore, crucial for raising the intelligence level of SLAM systems and can further increase the positioning accuracy and resilience of visual SLAM systems. It also offers fresh concepts for feature extraction and dynamic object detection.

### 3.4. Direct Method-Based Fusion Method

By utilizing the image’s pixel information and immediately predicting pose from the brightness values, this technique combines LiDAR and visual sensors. The direct approach of fusion performs especially well in dynamic contexts since it can better manage image alterations.

This method achieves the fusion of LiDAR and visual sensors by using the pixel information of the image, directly estimating the pose based on the brightness values of the image, and estimating the pose by minimizing the brightness differences of the image. Specifically, given the current frame image and LiDAR measurement values, the relationship between the image brightness values and the LiDAR data is utilized to infer the update of the camera pose. Lighting conditions can have a significant impact on the pose estimation in LiDAR and visual sensor fusion SLAM. For example, dynamic lighting changes (such as moving from a bright environment to a dark area) can lead to inconsistencies in image brightness, which will reduce the reliability of the direct method. Uneven lighting (such as the coexistence of shadows and direct light) makes it impossible for brightness values to effectively reflect scene features, which will further affect the matching accuracy. To address these challenges, combining lighting normalization technology with lighting invariance features would be a good choice, which will improve the robustness of the algorithm in variable environments. Therefore, the changes in lighting conditions must be fully considered in the direct method to optimize the accuracy and stability of pose estimation. At the same time, due to the better handling of changes in the image by direct method fusion, its performance in dynamic environments is particularly outstanding.

To improve the accuracy and robustness of the system, Jakob Engel et al. (2018) proposed a direct and sparse visual odometry method called direct sparse odometry, or DSO. This method can efficiently optimize all model parameters and reconstruct all pixels. It achieves this by optimizing the photometric error defined on the neighboring pixels of each point in real-time processing [34,35]. The fusion method based on tight coupling not only optimizes all model parameters but also accelerates the initialization process of the system by adding a stereo camera, thus directly minimizing the photometric error in dynamic multi-view stereo vision, thereby improving the system’s robustness and accuracy [36].

**Figure 6:**
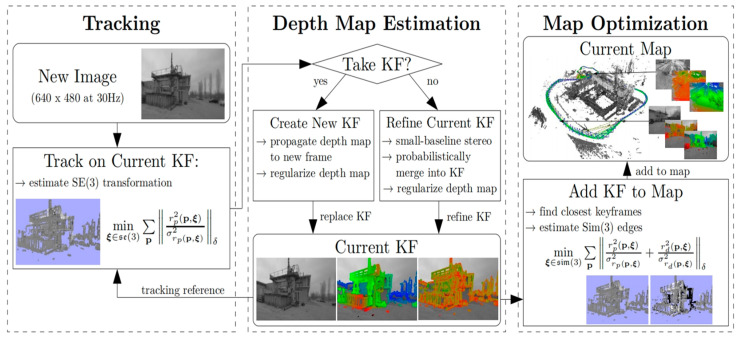
The entire LSD-SLAM algorithm system framework diagram [38].

In order to increase the system’s dependability, Bescos et al. (2018) introduced the DynaSLAM system, which improves SLAM’s performance in dynamic settings by mending the backdrop and recognizing dynamic objects, demonstrating the efficiency of the direct technique in managing dynamic obstacles [37]. Despite its notable advancements in tracking and mapping in dynamic scenarios, DynaSLAM still has limitations in regard to computing complexity, map updating capability, and light adaption, particularly exhibiting considerable fragility in settings with substantial light variations. Additionally, the method might not correctly separate and identify objects due to the light difference between dynamic objects and static backdrops, which could compromise the stability of posture estimation. Therefore, it is possible to improve the system’s light adaptation ability by introducing deep-learning technology, optimizing the algorithm structure, and improving the map-updating mechanism in order to effectively improve DynaSLAM’s performance and make it more adaptable to practical applications in complex dynamic environments. The direct method’s potential in large-scale settings is further demonstrated by the LSD-SLAM (large-scale direct monocular SLAM) approach. It overcomes the drawbacks of current direct approaches and builds a consistent environmental map in real-time settings by employing a novel tracking technique that corrects scale drift and increases pose estimation accuracy. Figure 6 is a diagram of the system’s framework. In real-time pose estimation, high-precision pose information is obtained by the direct image alignment method, and the pose map of keyframes and the semi-dense depth maps associated with these keyframes are generated by reconstructing the three-dimensional environment in real time. Simultaneously, the tracking mechanism successfully incorporates the noise of the predicted depth values through probabilistic approaches, improving the system’s capacity to manage depth estimation uncertainty [38]. However, there are still drawbacks, including inadequate texturing, excessive computational cost, and data association problems, even with LSD-SLAM’s strong performance in large-scale monocular SLAM. For instance, LSD-SLAM’s feature extraction and matching skills deteriorate on uniform or low-texture surfaces (like blank walls or floors), which results in unstable posture estimation. In these situations, it is challenging for the algorithm to gather enough useful data for precise positioning and mapping. By adding light-invariant features, integrating deep-learning technology, increasing computational efficiency, and improving the dynamic object-processing capability, future research can successfully boost LSD-SLAM’s performance and make it more resilient and adaptive in complex environments.

Therefore, the direct technique’s ability to manage correlation between various data kinds in a more systematic manner is its major benefit over the conventional feature-based fusion method. Applications with high real-time requirements benefit from the direct method’s ability to save time on feature extraction and matching by using pixel intensity information directly for motion estimation. Additionally, because the direct method does not rely on feature point matching, it reduces the accumulation of errors caused by the loss or incorrect matching of feature points, improving the system’s robustness and real-time performance.

These techniques work together because they can enhance and supplement one another to improve SLAM performance:

(1) In addition to being complementary, multimodal data fusion techniques create a broad framework that can offer an integrated platform for many sensors. This includes feature-based, direct, and semantic aid techniques, all of which can be used in multimodal SLAM to make use of the benefits of many sensors and enhance system performance;
(2) Feature-based fusion approaches provide a versatile way to manage feature points, providing extra pose constraints through feature matching, which are useful for merging with other methods to create a more efficient SLAM. This technique offers a versatile approach to managing feature points that are part of the feature layer fusion level. Through feature matching, this degree of fusion can offer extra pose restrictions, hence enhancing the SLAM system’s positioning precision and map-building capabilities in many contexts. It can be used with other techniques to produce a SLAM that is more effective. Combining these techniques allows us to create SLAM systems that are more robust, adaptable, and effective for a range of application scenarios and surroundings with various needs. The algorithm has more alternatives thanks to multimodal fusion, which helps it function well in a variety of challenging situations. By integrating these methodologies, we may create more powerful, adaptable, and efficient SLAM systems for diverse types of surroundings and application scenarios with different needs. The algorithm has more alternatives thanks to multimodal fusion, which helps it function well in a variety of challenging situations;
(3) By providing high-level contextual information, semantic information-assisted SLAM techniques can aid the system in comprehending the scene, particularly in complex environments. They can also be used in conjunction with feature-based and direct techniques to improve positioning accuracy and system stability;
(4) In order to create a hybrid approach, the direct technique combines features with semantic information. By optimizing the original data using these sensible techniques and using the depth information of the direct method to improve feature matching, it increases the SLAM system’s accuracy and resilience in a variety of settings.

In conclusion, SLAM technology, which combines vision and LiDAR, has demonstrated significant promise in handling dynamic and complex situations. These experiments offer fresh concepts for upcoming applications in challenging conditions, in addition to proving that combining LiDAR and vision is feasible. The goal of the research on the integration of LiDAR and vision sensors is to improve the system’s resilience and versatility, which are crucial in modern applications.

## 4. Main Research Achievements and Evaluation Analysis

The accuracy, resilience, real-time performance, and adaptability of laser and visual fusion SLAM technology are being studied by an increasing number of academics as the current research progresses. This will help to advance the technology’s use in complex environments and significantly increase its adaptability. Deep learning, for instance, has significantly enhanced performance in a number of areas, including deep estimation, semantic comprehension, data association, and optimization. It has also made feature extraction and matching more efficient. The research findings can also be validated and evaluated in real-world applications, which advances multimodal fusion and theoretical research while improving the performance of laser and visual fusion SLAM. At the same time, it offers fresh approaches to the problems that the existing laser and visual fusion SLAM faces in dynamic environments.

### 4.1. Analysis of Accuracy and Robustness

First and foremost, accuracy pertains to the system’s positioning and mapping precision, which is a crucial component of SLAM system assessment. Recent studies have demonstrated that combining several sensors can greatly enhance the SLAM system’s location precision and environment reconstruction quality [39]. The SLAM system’s various sensors were analyzed and compared, and it was discovered that effective environment reconstruction quality and positioning accuracy could be maintained in many situations, particularly when combined with optical sensors [40]. The current SLAM system framework algorithm has also been enhanced at the same time. Additionally, optimization can greatly increase the system’s accuracy and efficiency. For instance, a new key area extraction technique called ROI-cloud (region of interest cloud) was presented by Zhou et al. (2020). It increases robustness in complicated contexts, effectively predicts the distribution of important areas, and introduces a non-parametric particle-filtering approach. To increase the matching algorithm’s speed and accuracy, redundant and dynamic point clouds are eliminated [41]. In addition, a matching strategy based on intensity values is introduced to incorporate intensity into the cost function, which greatly improves the accuracy of the corresponding search for edge points and plane points. [42]. Additionally, analyzing deformed scenes with the DeSLAM (Deep SLAM) technique greatly increases accuracy. By merging template shape and non-rigid structure motion technology, it improves accuracy by tracking the camera pose and the deformation of the observation map in real time [43].

Furthermore, Mostafa Ahmadi et al. (2023) conducted a thorough analysis of various approaches and proposed a system based on HDPV-SLAM (hierarchical deep perception for visual SLAM) that adopts an improved two-stage adaptive refinement scheme (PanoDARS) by introducing a depth estimation module and a depth correlation module. This greatly improves the positioning and map construction accuracy of mobile mapping systems based on oblique multi-line lidar and panoramic cameras [44]. This significant performance improvement demonstrates not only the efficacy of the fusion method but also its potential for practical applications. Thus, increasing accuracy can help the system make more accurate positional estimates for itself, which can effectively reduce positioning mistakes and the accumulation of errors, further enhancing the map’s accuracy and consistency to handle increasingly complicated situations.

Second, robustness describes how consistently the SLAM system perceives its surroundings despite interference or impediments. The CAT-SLAM (collaborative autonomous tracking SLAM) system was introduced by Maddern et al. (2011) to provide stable map creation in various real settings. Its probabilistic local filtering improves the system’s robustness in dynamic environments while maintaining accuracy [45]. Furthermore, Zou et al. (2013) employed the CoSLAM (cooperative SLAM) technique, which combines the trajectories of static backdrop points with dynamic foreground points, to effectively accomplish visual SLAM in a dynamic environment by collaborating with many independent cameras. The system’s resilience is increased through attitude mapping and estimation [46]. A DOT system that combines instance segmentation with multiple views was developed by Ballester et al. (2021) as an alternative to using data from many independent cameras to boost the system’s reliability. Dynamic object tracking takes advantage of geometry and ORB attributes [47]. This technique can greatly increase SLAM’s accuracy and resilience in dynamic settings. While fusion technology research continues to advance, scientists have also made the initial discovery of the present LiDAR–camera fusion. By dividing the LiDAR and camera modalities into two separate streams, an underappreciated drawback of the method—namely, its reliance on the LiDAR input—is thereby addressed, greatly improving the system’s robustness to LiDAR failure scenarios. Pyojin Kim et al. (2023) developed a linear SLAM technique to handle increasingly complicated scenarios. By adding orthogonal plane features to the global model, the system effectively accomplished camera position prediction in dynamic environments. In addition to simplifying the nonlinear optimization issue, this approach increases the system’s robustness, enabling it to function well in a range of settings [48].

As a result, increasing the SLAM system’s robustness not only makes it more adaptable in complex settings but also better handles data association problems within the system, lowers matching errors brought on by environmental changes or sensor errors [49], and increases adaptability in complex environments.

### 4.2. Analysis of Real-Time and Adaptability

One of the key metrics for assessing SLAM systems is real-time performance, particularly in application settings where a quick reaction is necessary. In order to assess real-time performance, Sturm et al. (2012) developed a standardized benchmark test for RGB-D SLAM systems early on. This test provided various real data sequences with temporal synchronization [50]. In order to update the map and positioning information, Ma et al. (2016) proposed a real-time RGB-D SLAM system that can align and fuse the current frame (a real-time captured image) with previously stored keyframes (important reference images). Additionally, the system can align the current frame to known plane models to enhance the accuracy of the map and the understanding of the surrounding environment [51]. Simultaneously, SLAM’s ability to adapt to complicated settings will also be crucial. By implementing mobile consistency-checking techniques and semantic segmentation, Yu et al. (2018)’s DS-SLAM system effectively mitigated the effect of dynamic objects on location accuracy [52]. Through the cooperation of five parallel threads, the system successfully managed the impact of dynamic objects, attaining high-precision trajectory prediction and exhibiting significant adaptability in highly dynamic settings.

Fabian Blöchliger et al. (2018) proposed a novel Topomap framework to reduce computation time and improve the efficiency of global path planning. This framework converts sparse visual SLAM maps into 3D topological maps, which significantly reduces computation time and improves system real-time performance [53]. This makes it easier for mobile robots to navigate in large-scale semi-structured environments. Tristan Laidlow et al. (2019) used deep learning to create rich depth maps in order to guarantee the high stability of the SLAM system in a changing environment. This allowed for the quick reconstruction of intricate scenes and further enhanced the system’s resilience and adaptability [54]. A new comprehensive RGB-D benchmark dataset, CoRBS, was proposed by Wasenmüller et al. (2023) to improve system robustness. It combines RGB-D SLAM benchmarks with real depth, color data, camera trajectories, and scene geometry. This allows for independent evaluation of the positioning and mapping components of the SLAM system, further encouraging real-time evaluation of the SLAM system in dynamic environments [55].

As a result, the accuracy, adaptability, and robustness of the present SLAM research are constantly being improved, and new techniques are also encouraging the field’s growth toward greater precision and a larger range of application scenarios.

### 4.3. Evaluation of SLAM’s Performance in Difficult Surroundings

#### 4.3.1. Evaluation of SLAM’s Performance in Dynamic Settings

Researchers have started looking into the constraints of SLAM in dynamic environments as a result of the ongoing advancements in lidar and vision fusion SLAM technology [56]. Static environmental assumptions are typically the foundation of traditional SLAM technology. In using object knowledge for 3D SLAM, Salas-Moreno et al. (2013)’s early SLAM++ greatly enhanced the system’s performance in complicated situations. The system can successfully carry out real-time 3D object detection and tracking by introducing the idea of an object graph, leading to improved description and prediction capabilities in dynamic scenarios [57]. This technique must, however, offer a geometric model of the object, create an object database beforehand, and only detect objects that are present in the database. It also requires a prior structure, which assumes that the scene is static and that all of the objects are on the same plane (ground). The effectiveness of several visual SLAM techniques in uniform indoor settings was examined by Ibragimov and Afanasyev (2017), who discovered that the combination of vision and lidar can greatly increase robustness in dynamic scenarios [58]. At the same time, by skillfully mixing the two, the accuracy of system location in dynamic situations can also be greatly increased by using sensor data, semantic segmentation, and dynamic object consistency-checking techniques.

**Figure 7:**
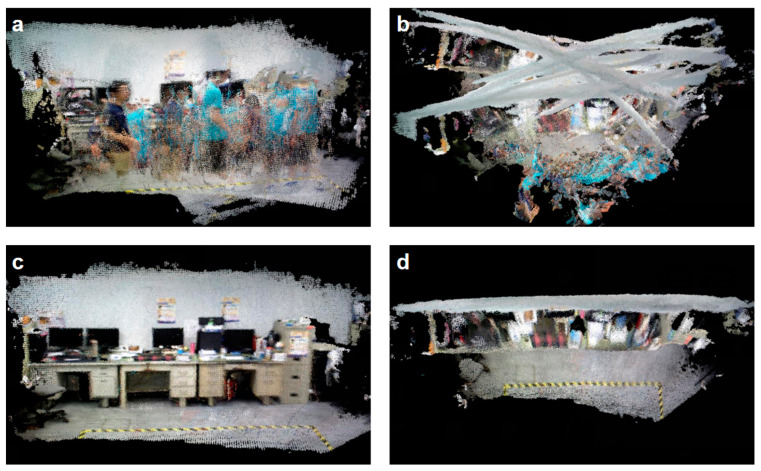
The maps produced by the original ORB-SLAM system. (**a**) System-generated map for ORB-SLAM-front view; (**b**) System-generated map for ORB-SLAM-vertical view; (**c**) ORB-SLAM with Sun et al.’s approach-front view; (**d**) ORB-SLAM with Sun et al.’s approach-vertical view [60].

Furthermore, the combination of LiDAR and vision offers fresh approaches to challenges in dynamic environments. To enhance the SLAM systems’ performance in dynamic situations, researchers have recently started looking into integrating deep learning and semantic information. For instance, the DynaSLAM system, created by Bescos et al. (2018), outperforms conventional SLAM techniques in terms of accuracy by using dynamic object detection and background filling to improve the robustness of dynamic scenes and function with monocular, stereo, and RGB-D visual camera configurations [59]. The system can still maintain high-precision map creation while improving the SLAM system’s flexibility in complicated situations by using deep-learning technology and multi-view geometry to detect moving objects. The maps produced by the original ORB-SLAM system are displayed as in Figure 7. The ORB-SLAM system can create the accurate map and effectively illustrate the detrimental effects of moving items in this dynamic environment by using the technique suggested by Sun et al. (2018) [60].

For instance, in real-world scenarios, fast-moving automobiles can be regarded as background elements in urban street scenes by the original ORB-SLAM, which could result in an inaccurate camera position estimate. Through depth information, the enhanced algorithm can rapidly determine an automobile’s dynamic properties, thereby removing it from feature matching. Likewise, in indoor settings, a person’s movement may alter the initial algorithm, resulting in erroneous map updates. In order to preserve the stability of the static environment, the enhanced algorithm can recognize this person’s movement trajectory and exclude pertinent information during posture optimization.

These studies demonstrate that, in order to adapt to various application circumstances, SLAM systems for dynamic environments must not only take algorithm efficacy into account but also attain system design flexibility. By analyzing RGB-D picture streams in real time to create scene-specific implicit 3D models, the iMAP technique effectively uses dynamic information to illustrate the use of multi-layer perceptrons (MLP) in real-time SLAM. This cutting-edge processing technique demonstrates the adaptability and promise of SLAM systems in dynamic environments.

In conclusion, the LiDAR and vision fusion SLAM system has demonstrated good performance in dynamic situations [61], particularly the approach that combines LiDAR and vision fusion SLAM with semantic reconstruction. It not only shows how this technique may greatly increase positioning accuracy in intricate settings but also amply validates the need for integrating LiDAR and vision sensors in dynamic environments.

#### 4.3.2. SLAM Performance in Situations with Limited Features

LiDAR’s performance has been greatly impacted in dynamic situations with few features. Thus, by incorporating scene layout knowledge, researchers have improved the state estimation and dense mapping capabilities in low-texture environments, thereby increasing the robustness in low-texture environments [62]. Deschaud (2018) showed the promise of LiDAR in dynamic situations by demonstrating a low-drift SLAM approach based on 3D scanning that reached a drift rate of 0.40% (i.e., 16 m of drift after 4 km) without loop closure [63]. To lessen their reliance on static world assumption, these techniques must nonetheless take into account how LiDAR data changes in dynamic situations. For instance, by combining visual information, Xu et al. (2018) presented a LiDAR-based SLAM technique that increases location accuracy in feature-scarce situations [64]. By combining the geometric features of points and lines, Albert Pumarola et al. (2017) developed the PL-SLAM method, which can efficiently perform SLAM in low-texture scenes. This improves the system’s state estimation and dense mapping capabilities in mapping [65].

In summary, advancements in laser radar and visual fusion SLAM technology have encouraged the modernization and refinement of different fusion algorithms in the current state of multi-domain SLAM technology. The development of multimodal SLAM, particularly the combination of laser radar and visual SLAM technology, has spurred new research avenues since the early iterations of basic sensor-matching algorithms. While SLAM technology currently exhibits great promise in complex environments, a more thorough investigation is still required to fully understand the system’s overall limitations.

## 5. Limitations and Trends in the Development of Current Research

### 5.1. The Limitations of the Research

Although there have been certain advancements in the SLAM technology that combines LiDAR and vision, the existing technology still faces significant challenges in terms of environmental adaptability and data association, among other aspects. Especially, there are still significant limitations in dealing with complex environments, consuming computational resources, and environmental adaptability. Future research needs to focus on these challenges, and these limitations also indicate the direction for future research.

(1) Handling of dynamic environments

While some studies have proposed solutions for dynamic environments, like DynaSLAM II and DS-SLAM, most SLAM systems still struggle to maintain high accuracy and robustness in highly dynamic environments, particularly when dealing with large-scale dynamic scenes and fast-moving objects [66]. Even with major advancements, some research notes that SLAM systems’ resilience in particular environmental circumstances is still problematic. When working with dynamic settings, Tete Ji et al. (2021) observed that current visual SLAM systems are likely to be constrained by high processing costs [67]. The effectiveness of existing approaches is often influenced by the interference of moving objects on environmental perception, restricting their application in real-world settings and necessitating additional refinement in large-scale dynamic scenes [68]. Effectively detecting and separating dynamic objects from static backgrounds is still a pressing difficulty in real-world applications, despite several researchers‘ attempts to enhance dynamic object-processing capabilities by integrating deep learning [69]. Current SLAM systems are still not sufficiently robust in dynamic situations. When dealing with unknown dynamic objects, the majority of current approaches frequently suffer from a reduction in positioning accuracy or map construction failure.

(2) Dependency on the sensors

The characteristics and limitations of different sensors will affect the fusion effect. How to select the appropriate sensor under different environmental conditions and optimize its fusion strategy are still challenges [70]. Most existing research focuses on the data processing of a single sensor, lacking effective fusion strategies. For example, Burgard et al. (2009) have already compared different SLAM algorithms and also emphasized the application of graph structures, but there is a lack of in-depth discussion on the specific implementation of sensor data fusion [71].

The fusion and processing of multi-sensor data are the primary challenges in the application of laser and visual fusion SLAM technologies. Data dependency in complex environments is still a major problem, as demonstrated by LVI-SAM (lidar visual-inertial odometry via smoothing and mapping; T. Shan et al., 2021), which has achieved tight coupling between LiDAR and visual–inertial odometry but still runs the risk of performance degradation in feature-poor environments [72]. Regarding data dependency, the majority of SLAM techniques rely on high-quality sensor data and favorable environmental circumstances to function well. However, in many practical circumstances, the acquisition and processing of data often meet issues such as noise, occlusion, and changes in lighting, which will have a negative impact on the stability and dependability of the SLAM system [73].

(3) Complexity of computation

As algorithm complexity rises, many fusion SLAM systems encounter a conflict between computer resources and processing speed. The current research is focused on how to balance both to obtain effective real-time performance [74]. The current deep-learning-based SLAM techniques are quite computationally intensive, which significantly reduces their usefulness. For instance, Edgar Sucar et al.’s iMAP (incremental mapping and positioning) method from 2021 has revolutionized efficiency, but it still necessitates a real-time training process and quite an intricate network topology, which increases the processing demands [75]. However, in complicated contexts, these methods’ accuracy will also be impacted.

(4) Adaptability to the environment

The effectiveness of the existing SLAM systems is frequently impacted by the interference of moving objects on visual characteristics, and they are not very flexible in complicated situations [76]. Reliable map generation and placement skills are frequently lacking in current approaches [77]. While visual SLAM continues to experience difficulties in situations like shifting lighting and interference from moving objects, laser radar-based SLAM is limited in some application scenarios due to its cost and equipment restrictions [78].

Furthermore, the feature-based fusion approach has drawbacks as well. The extraction and matching of feature points are crucial to this approach, and in complicated situations, feature point extraction may result in reduced performance [79]. According to Yan et al. (2024), classical VSLAM primarily uses scene features, namely point features, which might lead to mapping failure in texture-sparse situations, since there is insufficient feature information [80]. Real-time performance will be impacted, particularly when computational resources are scarce, by the computationally demanding feature point extraction and matching process.

As a result, in complex contexts, SLAM technology still faces difficulties with system environment adaptation. Even while some research has tried to use deep learning and semantic segmentation to enhance the tracking and detection of dynamic objects, it is still crucial to figure out how to successfully incorporate semantic information with conventional SLAM frameworks [81].

### 5.2. Trends in Research

New opportunities for enhancing system performance have arisen due to the ongoing development of technologies like multimodal data fusion, deep learning, and semantic information integration. This has resulted in the ongoing emergence of new trends and challenges, particularly with regard to technological innovation and application expansion. Multimodal sensor fusion, algorithm optimization, real-time performance enhancement, and application scenario expansion are the four primary areas of the research trends.

(1) Fusion of many sensors

By combining LiDAR with optical sensors, researchers have now greatly increased location accuracy. They have also integrated motion consistency checks and semantic segmentation in dynamic surroundings, proving the usefulness of fusion technology in dynamic situations. In order to fully benefit from both sensors, the system’s performance in complicated settings is enhanced by using depth information to boost environmental awareness and map construction capabilities. This fusion approach greatly increases the capacity to manage dynamic impediments while also increasing accuracy in complex contexts.

**Figure 8:**
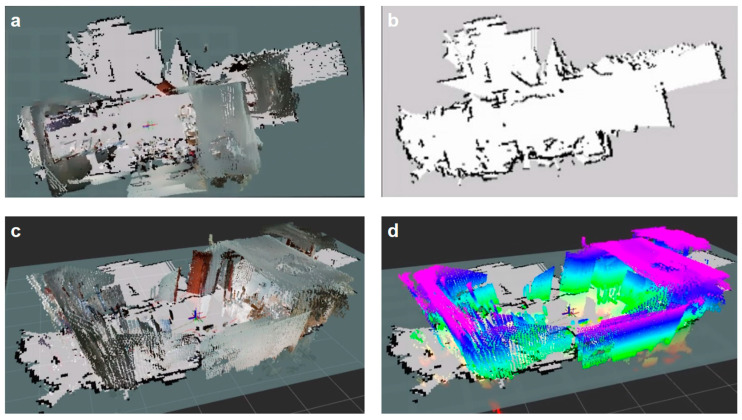
The suggested environment perception system based on LiDAR and vision. (**a**) The map in top perspective; (**b**) A navigational two-dimensional grid map; (**c**) Side view of the map; (**d**) Three-dimensional point cloud map; SLAM technology illustration using multi-sensor fusion.

In order to overcome the constraints of traditional sensors in particular situations, researchers can investigate different kinds of sensor combinations in the future and strengthen the fusion of new sensors. This will give SLAM systems more perception information, which will improve overall performance [82]. For instance, the suggested environment perception system based on LiDAR and vision offers a preliminary investigation of multi-sensor fusion through the combined use of LiDAR and infrared sensors, as well as sound sensors [83], as shown in Figure 8. Further investigation into the data fusion techniques of various sensors can lead to improved positioning accuracy and resilience, as well as a more thorough comprehension of the surroundings [84].

The following are some benefits of the fusion scheme. Fusion algorithms can be used to increase surveying accuracy when laser and visual information are regularly tracked. Laser SLAM placement can produce continuous results when visual tracking is unsuccessful. In addition, a two-dimensional laser can make up for the depth camera’s narrow range of vision, improving system navigation safety [83].

(2) Optimization of algorithms

As deep-learning technology advances quickly, more and more SLAM system algorithms incorporate deep-learning models to improve data processing and feature extraction [85], which raises the precision of map creation and placement [86]. For instance, using neural networks greatly increases the environmental adaptability of SLAM systems while also improving their performance in difficult circumstances. One of them, active neural SLAM, was proposed by Devendra Singh Chaplot et al. (2020) and integrates learning modules with traditional path planning [87]. The SLAM field is moving toward more efficient algorithms and modularized systems as a result of Chen et al. (2021) using deep neural networks for loop detection in their OverlapNet [88] and DROID-SLAM (Z. Teed et al. 2021) using a recursive iterative method to update camera pose and pixel and depth information [89]. This leads to more accurate [90] environmental understanding and map generation [91] by increasing the system’s adaptability to input modalities and strengthening the resilience of system state estimation.

The optimization of current SLAM algorithms may be the main focus of future studies. For instance, current LiDAR and visual SLAM techniques mostly use a loose coupling approach and have not fully used the benefits of both. In order to increase the precision and dependability of state estimates, future studies should concentrate on how to obtain closer sensor fusion. In order to increase accuracy and speed up calculation, neural networks are also combined with conventional SLAM algorithms [92]. Furthermore, the next phase of the study might concentrate on developing more effective algorithms that would further enhance performance in intricate settings while striking a better balance between accuracy and real-time performance [93], and simultaneously, improve intelligence and learning by integrating reinforcement-learning technology and investigating adaptive SLAM systems that may dynamically modify algorithm parameters in response to changes in the environment, increasing the system’s stability and adaptability [94].

(3) Improvement of performance in real time

Current SLAM research trends aim to improve real-time processing capabilities to adjust to changing environments, in addition to increasing algorithm accuracy and robustness [95]. Thus, for real-time applications in dynamic situations, effective data bundle adjustment (bundle adjustment) in SLAM algorithms is very crucial [96]. Researchers are dedicated to improving the computational efficiency of SLAM algorithms in order to satisfy the demands of real-time applications. By using explicit volume representation techniques, the SplaTAM framework created by Nikhil Keetha et al. (2024) improves the real-time performance of the SLAM system by achieving high-fidelity reconstruction from a single uncalibrated RGB-D camera [97]. To enhance the system’s overall performance, future studies can investigate how to include dynamic object detection and tracking algorithms in real-time SLAM. To lessen their impact on the environmental map’s creation, dynamic items will need to be identified and modeled.

(4) Extension of application scenarios

As technology develops, the range of applications for laser radar and visual fusion SLAM keeps growing, ranging from conventional interior settings to intricate dynamic outdoor scenery and even to cutting-edge domains like autonomous driving and augmented reality. Future studies can concentrate on applying SLAM technology to real-world situations, particularly ones with a lot of dynamism and ambiguity. It improves the system’s resilience and interpretability while bolstering users’ comprehension and confidence in the SLAM system’s decision-making process. Furthermore, for SLAM systems to handle a variety of real-world application scenarios, they must possess enhanced environmental adaptability and self-learning capabilities [98]. To maintain effective navigation and positioning capabilities, for instance, SLAM systems must be able to adjust to environmental changes in real time in dynamic environments like agriculture [99]. Additionally, in dynamic environments, SLAM systems must be able to manage the impact of dynamic objects to enhance system performance.

## 6. Conclusions

For visual fusion with lidar, by integrating the benefits of multimodal sensors, SLAM technology efficiently makes up for the shortcomings of a single sensor in complex environments and has emerged as a crucial tool for enhancing autonomous positioning and mapping capabilities. After incorporating the artificial intelligence algorithm framework, the fusion SLAM system has improved accuracy and robustness in a variety of challenging scenarios by combining the benefits of both. Consequently, the SLAM technique, which combines visual and lidar data, has steadily grown in popularity as a research area.

Despite showing a promising development trend, SLAM technology that combines vision and LiDAR still has certain drawbacks. First of all, existing algorithms are encountering greater real-time and accuracy issues due to the complexity of application cases. Consequently, we should optimize the framework algorithms in the upcoming research phase to minimize the usage of computational resources and, to the greatest extent feasible, enhance the system’s real-time and environmental adaptability; Second, we should investigate ways to propose more efficient fusion methods without compromising performance while optimizing the algorithms to save computing resources. The research focus should be on the realization of low-latency, high-efficiency multimodal data fusion, which will effectively reduce the SLAM system’s energy consumption and computational burden while also improving the system’s accuracy and real-time. At the level of data fusion algorithms, effective data fusion can be implemented by adopting advanced state estimation frameworks, such as information filtering and particle filtering, to efficiently handle the heterogeneity of laser and visual data. Second, using deep-learning technology for feature extraction and multimodal learning can also greatly enhance the robustness of feature matching. At the same time, sparse optimization algorithms and incremental map update strategies are designed to reduce computational complexity and latency. Finally, in subsequent research, we will achieve low-latency and high-efficiency multimodal data processing by establishing a unified state estimation framework and adaptive fusion strategy, thus ensuring system performance while significantly improving the accuracy of the system’s real-time positioning. It is believed that this method will provide a solid foundation for SLAM applications in more complex and dynamic environments. Lastly, LiDAR’s high cost prevents it from being widely used in low-cost application scenarios, which will also encourage academics to look into less expensive hardware options or create effective algorithms that work with low-cost hardware.

To sum up, the SLAM technique that combines vision and LiDAR offers a lot of potential for real-world uses. Multi-sensor fusion, more adaptation to changing settings, and widespread use of deep-learning technologies should be the main areas of future development to increase the SLAM system’s resilience and adaptability. In addition to encouraging the ongoing development and expansion of technology, this will result in more creative solutions for domains like autonomous driving and robot navigation.

## Data Availability

Data are contained within the article.
